# Understanding consumer impulse buying in livestreaming commerce: The product involvement perspective

**DOI:** 10.3389/fpsyg.2023.1104349

**Published:** 2023-03-15

**Authors:** Xiaoxiao Gong, Xuetao Jiang

**Affiliations:** ^1^School of Management, Guizhou University, Guiyang, China; ^2^Karst Region Development Strategy Research Center, Guizhou University, Guiyang, China

**Keywords:** livestreaming commerce, impulse buying, product cognitive involvement, product affective involvement, product attributes

## Abstract

The rapid development of livestreaming commerce has received widespread attention from both theoretical and practical circles. However, relatively few studies have been conducted from a product perspective, and even fewer studies have analyzed product characteristics influencing consumers’ impulse buying based on product-involvement theory. Grounded on product involvement theory, this study proposed a theoretical research model and empirically tested the model using online survey data collected from 504 livestreaming consumers in China. The results showed that functional value for money, perceived product quality, perceived product scarcity, instant feedback on product information, and perceived product knowledge of streamers can drive product cognitive and affective involvement, which, in turn, induce the consumer-felt urge to buy impulsively and engage in impulse buying behavior. However, the functionality of product design can only affect the product cognitive involvement, not the affective involvement. Implications for research and practice are discussed.

## 1. Introduction

The continuous advancement of communication technology and the upgrading of mobile devices provide the basis for the development of existing mature social commerce platforms for livestreaming commerce (Kang et al., 2021). In the past 4 years, consumers have actively engaged in live shopping through platforms such as Taobao, TikTok, and Mushroom Street (Chen et al., 2022; Li et al., 2021). According to the 50th report of the China Internet Network Information Center (CNNIC), as of June 2022, China’s livestreaming users have reached 716 million, of which 469 million were livestreaming commerce users, accounting for 68.1% of the overall netizens. Livestreaming commerce has attracted continued attention from enterprises as an essential marketing channel and a driving force for e-commerce sales growth (Ma, 2021). Livestreaming commerce is a marketing behavior in which streamers use web terminals such as computers and mobile phones to promote products *via* livestream and provide shopping links to facilitate transactions in a short period of time (Lee and Chen, 2021). Through livestreaming commerce, streamers can show product details from different perspectives, interact with customers in real-time, and inform specific details such as logistics and delivery, after-sales returns, etc. As livestreaming is increasingly used to promote products to potential customers, the competition in the livestreaming commerce industry has become more intense (Zhang et al., 2021). Therefore, for companies to gain market advantage, it is crucial to explore how to effectively improve the purchase rate of customers in livestreaming commerce and facilitate transactions from livestreaming traffic.

Just as there is great interest in livestreaming from an industry standpoint, the academic community has also conducted numerous studies on live marketing, focusing mainly on user stickiness (Li et al., 2021), user engagement behavior (Hu et al., 2017; Hilvert-Bruce et al., 2018; Hu and Chaudhry, 2020; Kang et al., 2021; Singh et al., 2021), product purchase intention (Sun et al., 2019; Chen et al., 2022; Xu et al., 2020), and impulse buying (Cheng, 2020; Gong et al., 2020; Jiang and Cai, 2021; Lee and Chen, 2021; Ming et al., 2021). Following from existing research, this paper focuses on consumer impulse buying behavior in livestreaming commerce, a typical consumer behavior characterized by a spontaneous, irresistible, assertive, persistent, and immediate desire to purchase a product (Zheng et al., 2019).

Current research has examined the characteristics of livestreaming platforms (Gong et al., 2020), streamer characteristics (Park and Lin, 2020; Ma, 2021), social presence (Ming et al., 2021), and more, but there is still a lack of research from the perspective of commodity characteristics of livestreaming marketing. Therefore, to address the gaps in the existing literature, this study constructed a research framework on consumer impulse buying behavior in livestreaming commerce based on product involvement theory. Structural Equation Modeling (SEM) was used to explore the influencing factors of consumer impulse buying behavior from the product features perspective, and an artificial neural network (ANN) was used to explore the importance ranking of the significantly influential antecedent variables. The findings of this study help to enhance the effectiveness of livestreaming marketing, expand the application context of product involvement theory, reveal the deep-rooted mechanism in impulse buying behavior, and provide a reference for e-commerce enterprises to use livestreaming for marketing activities effectively. To reach its objective, the study attempted to address the following research questions:

Q1. Which of the general and specific attributes of a product in livestreaming commerce affect consumers’ impulse buying?

Q2. What is the order of importance of the above factors?

The rest of the paper is organized as follows. In Section 2, we review theories about product involvement and present the main hypotheses of this study. Then, we present the research design, including the methodology, data collection process, and variable construction in Section 3. Next, SEM and ANN analysis and results are presented in Section 4. In the final section, the theoretical implications, managerial implications, and future research are summarized.

## 2. Theoretical model and research hypothesis

### 2.1. Product involvement theory

Involvement theory was first derived from the concept of self-involvement (Sherif and Cantril, 1947), which was proposed to explain one’s attitude towards accepting different ideas about an event. Krugman (1966) introduced the concept of involvement into consumer behavior research and then applied it to different domains. However, it is still mainly used to explain decision-making processes directed at consumers and is considered to be a motivational state, that is, the perceived personal importance in acquiring, consuming and depositing products (Celsi and Olson, 1988). It is a construct that uses the consumer’s cognitive style to explain their purchase behavior. Zaichkowsky (1985) broke involvement down into advertising involvement, product involvement, and purchase decision involvement. Of these, product involvement refers to the difference in the degree of importance and response of consumers to the differences in attributes offered by different products or brands. The proposed involvement-brand loyalty model argues that there are two states of involvement when consumers make purchases, namely product involvement and brand involvement, with product involvement referring to the level of interest in the product category (Mittal and Lee, 1989). A number of related studies on product involvement have since emerged, such as Huang et al. (2010) who pointed out that product characteristics such as perceived risk, price, symbolism, durability, pleasure, importance, brand, and time to purchase may influence the degree of online user involvement. Schuitema et al. (2013) proved that symbolic value, hedonic value, or the extent to which the product provides pleasure and evokes emotions associated with purchase and consumption are sources that trigger involvement. Further research has found that higher values of technological, hedonic, and symbolic differences in products increase the propensity to purchase among highly involved consumers (Chao et al., 2021).

Product involvement has been divided into product cognitive involvement and affective involvement (Perse, 1990; Jiang et al., 2010; Drossos et al., 2014; Kang et al., 2015; Faisal et al., 2020). Product cognitive involvement refers to the psychological response caused by the functional and utilitarian aspects of the product (Zaichkowsky, 1994; Drossos et al., 2014). This information, including inference and factual information, is the sort that consumers seek when forming attitudes and intentions about a product. Affective involvement is a psychological response based on feelings, emotions, and mood (Zaichkowsky, 1994; Drossos et al., 2014), which is caused by value expressions or emotional motivations (Perse, 1990; Celuch and Slama, 1998; Jiang et al., 2010). Studies have been conducted examining the effects of cognitive and affective product involvement on consumer behavior and advertising. For example, Faisal et al. (2020) examined the impact of design quality (i.e., font and aesthetic quality, information quality, navigation quality, and interactivity) on cognitive and affective involvement, which ultimately leads to persistent intention to use. Meanwhile, Drossos et al. (2014) have proven the impact of cognitive and affective product involvement on purchase intention in the context of mobile advertising.

Although studies have established and validated the cognitive-affective involvement dichotomy in offline marketing and online shopping contexts, these studies have not yet been conducted in the context of livestreaming commerce, whose real-time interaction and time-independent characteristics may differ from traditional or social commerce. Therefore, we applied product involvement theory to livestreaming commerce to explore its explanatory power in consumer impulse buying behavior.

### 2.2. Research hypothesis

#### 2.2.1. Functional value for money and product involvement

Functional value for money (price) is considered to be the utility derived from a comparison of benefits and costs (Sweeney and Soutar, 2001; Zhang et al., 2020), which is a concept of value for money, implying a greater monetary advantage over other options (Sheth et al., 1991). Related studies have used a similar concept of “monetary value” as the main driver of consumer choice. For example, Singh et al. (2021) proved that monetary value significantly impacts perceived value, and that perceived value provides easy access to services with the right monetary value. The impact of product price on the level of involvement of online users has been demonstrated (Huang et al., 2010). Specifically, at the perceived level, consumers enter more focused on the utility value of the product and more sensitive to its price/performance ratio. That is, if the product recommended by the streamer is value for money, it may lead to a higher level of consumer cognitive involvement. At the affective level, Houston (1978) believed that product complexity and product features, such as price, time, and place of consumption, directly contribute to contextual involvement. Therefore, it can also be inferred that the price of a product in the live room also leads to the affective involvement of consumers. Based on the above arguments, we proposed the following hypotheses:

*H1*-a: The functional value for money in livestreaming commerce positively influence product cognitive involvement.

*H1*-b: The functional value for money in livestreaming commerce positively influence product affective involvement.

#### 2.2.2. Perceived product quality and product involvement

Perceived product quality is the subjective perception or judgment of consumers about the overall excellence or superiority of a product (Chinomona et al., 2013; Chen et al., 2022). Consumers evaluate products based on perceived quality and expected performance (Slack et al., 2020). It has been shown that there is a positive relationship between product quality and consumer involvement (Tsiotsou, 2006), but most research thus far has demonstrated the effect of consumer involvement on perceived product quality (Tsiotsou, 2006; Campbell et al., 2014; Rokonuzzaman et al., 2020). Contrary to the findings of these studies, we argue that consumers are more likely to try out a product if they perceive it to be of higher quality. Specifically, product quality is an important part of consumers’ decision-making process (Rokonuzzaman et al., 2020), in that when watching a livestream, consumers perceive that the streamer is recommending a higher quality product which then makes the consumer feel the perceived involvement of utility. If consumers perceive higher product quality, this can also trigger usage emotions, as shown by Foroughi et al. (2016) who found that both core product quality and user performance are significantly associated with both negative and positive emotions of use. Based on the above arguments, we proposed the following hypotheses:

*H2*-a: Perceived product quality in livestreaming commerce positively influences product cognitive involvement.

*H2*-b: Perceived product quality in livestreaming commerce positively influences product affective involvement.

#### 2.2.3. Perceived product scarcity and product involvement

Perceived product scarcity is the lack of real or perceived goods and services available to consumers in the short term (e.g., due to being out of stock) or in the long term (e.g., due to legal restrictions; Hamilton et al., 2019). Research has shown that consumer perceptions can be influenced by the state of scarcity (Shah et al., 2015). When a product is difficult to purchase, the value of the product increases and consumers will want more (Akram et al., 2018), which will in turn increase the perceived involvement of the product. Meanwhile, product scarcity will increase the perceived value and consumer enjoyment of the product, creating positive value of the product in the consumer’s psyche. If the product is positioned as being available for a limited time only, this will make the consumer experience more enjoyable (Hamilton et al., 2019). Hence, the limited availability of a product will deepen the emotional involvement of consumers. Therefore, based on the above arguments, we proposed the following hypotheses:

*H3*-a: Perceived product scarcity in livestreaming commerce positively influences product cognitive involvement.

*H3*-b: Perceived product scarcity in livestreaming commerce positively influences product affective involvement.

#### 2.2.4. Functionality of product design and product involvement

Functionality of product design refers to the perception that reflects the consumer’s ability of the product to achieve its purpose (Homburg et al., 2015). It has been shown that perceived functionality is particularly important for online shopping as it prevents consumers from experiencing the product in its entirety (Spears and Yazdanparast, 2014). Product design features are reliable indicators of functional performance, and when a product appears to offer a high level of performance in terms of functionality, consumers form expectations about the performance of those features (Hoegg and Alba, 2011). Thus, the features that streamers recommend for product design during livestreaming will stimulate consumers to generate product awareness involvement. Product design also influences consumers’ evaluation of the product, and their immediate desire to own the product (Reimann et al., 2010; Homburg et al., 2015). We also infer that the features of the product design positively influence affective involvement. Therefore, based on the above arguments, we proposed the following hypotheses:

*H4*-a: Functionality of product design in livestreaming commerce affects product cognitive involvement.

*H4*-b: Functionality of product design in livestreaming commerce affects product affective involvement.

#### 2.2.5. Instant feedback on product information and product involvement

Instant feedback on product information refers to the extent to which there is no delay in the feedback to the communication (Steuer, 1992; Alba et al., 1997; Burgoon et al., 2002; Li and Peng, 2021). Thus, instant feedback of product information means that consumers can communicate in real time with the streamer, other users in the same livestream, and store customer service about product performance, price, logistics, and after-sales information. In livestreaming, the real-time and synchronized nature of the medium allows both the streamer and the user send or receive information without delay (Li and Peng, 2021), making it easier for consumers to become cognitively involved with the product. Accordingly, consumers may become less self-conscious and focus on the experience in livestreaming (Li and Peng, 2021). It has been shown that instantaneous information exchange is considered to be a key element of a personalized experience, allowing consumers to communicate their needs without the constraints of time and space (Buhalis and Amaranggana, 2015; Lei et al., 2020; Chen and Liao, 2022; Zheng et al., 2023a,b). Therefore, consumers become emotionally involved with the products recommended by the streamers in the livestream. Based on the above arguments, we proposed the following hypotheses:

*H5*-a: Instant feedback on product information in livestreaming commerce positively influences product cognitive involvement.

*H5*-b: Instant feedback on product information in livestreaming commerce positively influences product affective involvement.

#### 2.2.6. Perceived product knowledge of streamers and product involvement

Perceived product knowledge of streamers refers to consumers’ perception of the depth of the streamer’s product knowledge, including whether the streamer has in-depth knowledge of the product and can answer consumers’ questions about the product in a timely manner during the livestreaming (Agnihotri et al., 2009; Chen et al., 2022). Park and Moon (2003) proved that the correlation between consumers’ product involvement and objective product knowledge is higher for utilitarian products than for hedonic products, while the opposite is true for subjective product knowledge. This also provides evidence for product knowledge and product involvement. Further, if the streamer has product-related expertise that is effective in satisfying customers’ needs (Chen et al., 2022; Liao et al., 2023) it allows consumers to generate cognitive involvement in the usefulness of the product. Consumers’ increasing trust in the streamer allows them to generate affective involvement with the product being recommended by the streamer. Therefore, based on the above arguments, we proposed the following hypotheses:

*H6*-a: Perceived product knowledge of streamers in livestreaming commerce positively influences product cognitive involvement.

*H6*-b: Perceived product knowledge of streamers in livestreaming commerce positively influences product affective involvement.

#### 2.2.7. Product involvement and impulse buying

Previous studies have found that involvement affects brand attitude, purchase intention, advertising attitude, online shopping behavior factors, and more (Bosnjak et al., 2007; Huang et al., 2010; Yang, 2012). However, evidence on the association between involvement and impulse buying is limited (Drossos et al., 2014). Zhao et al. (2019) considered engagement, interaction, and pleasure as the three dimensions of product involvement, which increase consumers’ urge to engage, interact, gain pleasure and enjoyment, and indulge in shopping sprees. When the level of consumer product involvement is higher, the likelihood of impulsive buying behavior has also been shown to be higher (Liang, 2012). For example, Cengiz (2017) believed that consumers with higher purchase decision involvement would be more likely to spend impulsively, and this tendency is more pronounced in the case of popular clothing purchases. Chan et al. (2017) determined through a literature review that cognitive and affective involvement would act as core influences on impulse buying. Specifically, product cognitive involvement – where consumers perceive the usefulness of a product and immediately develop a desire to own it – is also more likely to result in the consumer purchasing an unwanted product being recommended by the streamer. The affective involvement of the product will cause consumers to feel more closely connected to the streamer-recommended product and generate an urge to buy impulsively and impulse buying behavior. Therefore, based on the above arguments, we proposed the following hypotheses:

*H7*-a: Product cognitive involvement in livestreaming commerce positively influences the consumer-felt urge to buy impulsively.

*H7*-b: Product affective involvement in livestreaming commerce positively influences the consumer-felt urge to buy impulsively.

*H8*-a: Product cognitive involvement in livestreaming commerce positively influences consumer impulse buying behavior.

*H8*-b: Product affective involvement in livestreaming commerce positively influences consumer impulse buying behavior.

#### 2.2.8. Felt urge to buy impulsively and impulse buying behavior

A series of studies explored the effect of the felt urge to buy impulsively on impulse buying behavior (Shen and Khalifa, 2012; Zhao et al., 2019; Lo et al., 2022). Although it has been argued that consumers may often develop impulse buying urge in response to external stimuli, there is a certain probability of conversion to impulse buying behavior, which does not necessarily mean that consumers will always act (Rook and Fisher, 1995). Nevertheless, Verhagen and Van Dolen (2011) suggest a significant positive relationship exists between these two factors, with purchase impulse providing the impetus for subsequent purchase behavior (Shen and Khalifa, 2012). Adelaar et al. (2003) also points out that impulse buying urge is sudden, powerful and irresistible and proves that felt urge to buy impulsively is positively related to impulse buying. Based on the suggestions of existing studies, this study proposes a positive relationship between the two of them in livestreaming commerce. That is, if consumers have a strong urge to buy a product, then they are more likely to impulsively buy that product while watching a live stream. Therefore, based on the above arguments, this paper proposes the following hypothesis:

*H9*: The consumer-felt urge to buy impulsively in livestreaming commerce positively influences impulse buying behavior.

**Figure 1:**
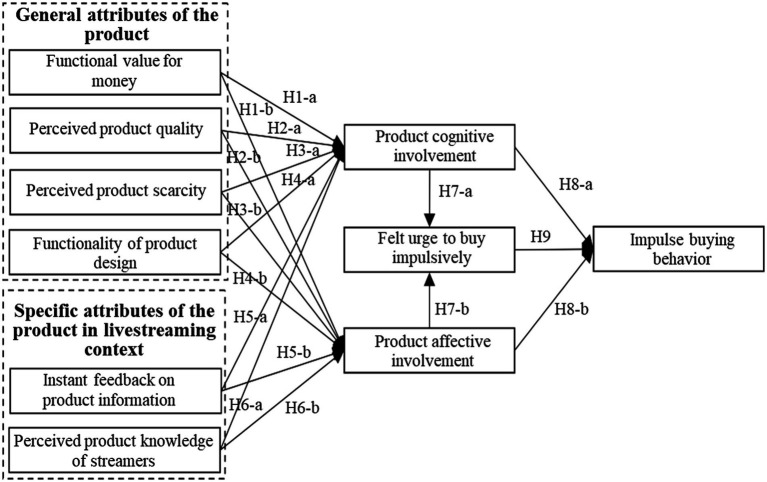
Research model.

Integrating the above theoretical analysis and hypothesis derivation, the research model of this paper is shown in Figure 1.

## 3. Research methodology

### 3.1. Questionnaire design

The questionnaire used in this study consisted of two parts. First, a survey on factors which influence impulse buying behavior in livestreaming commerce consumers, including the functional value for money (PRI), perceived product quality (PQ), perceived product scarcity (PST), functionality of product design (PDF), instant feedback on product information (IF), perceived product knowledge of streamers (KN), product cognitive involvement (CI), product affective involvement (AI), felt urge to buy impulsively (BI), and impulse buying behavior (IB). Second, sample demographic variables were collected, including gender, age, education level, monthly income level, and weekly viewing time. Regarding the design of the questionnaire, the measurement questions in the current study were adopted from existing studies and were modified appropriately according to the actual situation of livestreaming commerce consumers in China. The questionnaire was designed using reverse translation, expert review, and pre-research to revise the items to ensure that the semantics and logic were consistent with the Chinese context. The specific measurement variables and reference sources are shown in Table 1, and all items were rated using a 7-point Likert scale.

**Table 1:** Variable measures, factor loadings.

Variable	Measure item	Measurement entries	Factor load	Reference
Functional value for money	PRI1	I think the products recommended by the streamer during the livestream are reasonably priced.	0.968	Zhang et al. (2020)
	PRI2	I think the products recommended by the streamer on the livestream are good value for money.	0.972	
	PRI3	I think the products recommended by the streamers are economical.	0.972	
Perceived product quality	PQ1	I think the products recommended by the streamers will meet my needs.	0.974	Chen et al. (2022)
	PQ2	I think the quality of the products recommended by the streamers is as advertised.	0.975	
	PQ3	I think the overall performance of the product recommended by the streamer was excellent.	0.978	
Perceived product scarcity	PST1	When I shop live, I consider when the item will go off the shelf.	0.973	Akram et al. (2018)
	PST2	When I shop live, I worry about the limited time it is available to purchase.	0.970	
	PST3	When I shop live, I consider the limited quantity of that item.	0.973	
	PST4	When I shop live, I worry that the item is sold out.	0.970	
Functionality of product design	PDF1	I think it’s possible that the products recommended by the streamers will perform well.	0.968	Homburg et al. (2015)
	PDF2	I think the product recommended by the streamer seems to deliver what it is said to.	0.969	
	PDF3	I think the products recommended by the streamer when livestreaming seem to be functional.	0.973	
Instant feedback on product information	IF1	While watching a livestream, I can quickly send/receive information about the products recommended by the streamer.	0.973	Li and Peng (2021)
	IF2	While watching a livestream, I can immediately know what others (e.g., the streamer, other viewers, online customer service) think about the streamer’s recommended products.	0.989	
	IF3	While watching a livestream, I can immediately let others (e.g., the streamer, other viewers, online customer service) know what I think of the product.	0.969	
	IF4	While watching a livestream, I am able to receive quicker responses from others (e.g., streamer, other viewers, online customer service) about the product information I need.	0.969	
Perceived product knowledge of streamers	KN1	It feels like this streamer knows the product well	0.973	Chen et al. (2022)
	KN2	If I wanted to buy a product today, I would need to gather very little information to make an informed decision.	0.976	
	KN3	I have a lot of faith in the streamer’s ability to judge the quality of the product.	0.969	
Product cognitive involvement	CI1	I think the products recommended by the streamer are what I need.	0.975	Faisal et al. (2020)
	CI2	I think there is value in the products recommended by the streamer.	0.972	
	CI3	I think the products recommended by the streamer are relevant to me.	0.974	
Product affective involvement	AI1	I think the products recommended by the streamer are charming.	0.965	Faisal et al. (2020)
	AI2	I think the products recommended by the streamer are interesting.	0.966	
	AI3	I think the products recommended by the streamer are attractive.	0.957	
	AI4	I think the products recommended by the streamer are easy to make people vicarious.	0.964	
Felt urge to buy impulsively	BI1	The moment I see a streamer recommending a product (service) live, I want to own that product or reward the streamer immediately.	0.985	Beatty and Ferrell (1998)
	BI2	When I see a streamer recommending a product (service) live, I develop a strong desire to buy it or reward the streamer.	0.964	
	BI3	As soon as I saw the streamer recommending a product (service) live, I thought that it’s what I wanted.	0.963	
	BI4	I had not planned to buy the product (service) beforehand, but was tempted to buy it after seeing the streamer’s live recommendation.	0.958	
Impulse buying behavior	IB1	I purchased a product I did not originally intend to buy or purchased a virtual gift to reward the streamer.	0.953	Jones et al. (2003)
	IB2	I’ve noticed a lot of products I’ve recently purchased *via* livestreams are rarely used.	0.954	
	IB3	I do not think deeply when I buy these products when watching a livestream, or when I buy virtual gifts to reward the streamers.	0.952	
	IB4	When I decide to buy a product while watching a livestream, or to buy a virtual gift to reward the streamer, there’s something about it that’s hard to resist and I want to have it.	0.952	

### 3.2. Sample selection

This study recruited livestreaming consumers in mainland China as the data source, and participants given detailed information about the study objectives, operational procedures, expected benefits, and ethical considerations regarding participation when recruited. Due to the livestreaming behavior explored in this study, we considered the concentration of the sample source as well as the convenience of the study, and chose to distribute the questionnaire using the Credamo platform (Gong et al., 2020).^1^ A total of 603 questionnaires were returned. After eliminating invalid questionnaires, 504 valid questionnaires were obtained, with an effective rate of 83.6%. The demographic characteristics of the sample are shown in Table 2, where the frequencies are the number of respondents, and the percentages denote the portion of the total sample for a certain category of respondents.

**Table 2:** Composition distribution of the sample (*N* = 504).

Control variables	Specific options	Frequency	Percentages
Gender	Male	204	59.5
	Female	300	40.5
Age	0–18 years	1	0.2
	19–22 years	49	9.7
	23–28 years	186	36.9
	29–40 years	244	48.4
	Over 40 years old	24	4.8
Education level	Junior high school	6	1.2
	High school	20	4.0
	Post-secondary	51	10.1
	Undergraduate	383	76.0
	Master’s degree and above	44	8.7
Monthly income level	No income	8	1.6
	Under ￥500	2	0.4
	￥501–￥1,000	12	2.4
	￥1,001–￥,2000	23	4.6
	￥2,001–￥3,000	30	6.0
	￥3,001–￥5,000	74	14.7
	￥5,001–￥10,000	249	49.4
	￥10,001–￥20,000	93	18.5
	Over ￥20,000	13	2.6
Weekly viewing time	Almost none, less than 2 h	19	3.8
	Relatively few, 2–7 h (average of less than 1 h per day)	153	30.4
	Medium level, 7–21 h (average of 1–4 h per day)	304	60.3
	More than 21 h	28	5.6

### 3.3. Analytical approach

The study worked to validate the research hypotheses and identify the antecedents/predictors of impulse buying by livestreaming commerce consumers through a two-step analytical approach using Structural Equation Modeling (SEM) and ANN analysis.

Studies have been conducted to apply systematic SEM-ANN approaches in different scenarios such as mobile social media adoption (Li et al., 2019), mobile learning (Alhumaid et al., 2021), wearable payments (Lee et al., 2020), mobile payments (Leong et al., 2018), and ERP systems (Zabukovšek et al., 2019). Since the SEM approach uses statistical modeling only for linear models, it sometimes oversimplifies the complexity of the model when consumers make impulse buying. Therefore, the ANN analysis method can be used to test the linear and non-linear relationships between the factors that characterize the product and consumers’ impulse buying decisions, achieving more accurate predictions. In addition, utilizing a two-stage SEM-ANN approach as a predictive analytic method provides a further overall understanding and provides important methodological contributions through analytical perspectives.

## 4. Results

### 4.1. Partial least squares – Structural equation modeling

Statistical software including SPSS 25.0 and Smart PLS 3.2 were used for data analysis in this study. An examination of skewness and kurtosis showed that the data showed normal distribution. Similarly, *Z*-scores for all survey items were examined and found to be below the threshold limit of 3.29, indicating that there were no potential outliers in the data. The processed data were analyzed using a two-stage analysis. First, the measurement model was estimated to check for measurement attributes such as reliability and validity associated with the study. Second, a structural model was estimated to test the study hypotheses and draw appropriate conclusions.

This two-step approach ensured that there were valid and reliable measurement attributes between the study structures in order to map the structural relationships.

#### 4.1.1. Measurement model

##### 4.1.1.1. Reliability analysis

In this paper, the reliability of the questionnaire is measured by Cronbach’s α coefficient and Composite Reliability (CR), both of which are generally used to measure the degree of internal consistency of the observed and latent variables, respectively. As shown in Table 3, the Cronbach’s α and CR values of the questionnaire used in this study were calculated to be above 0.9, which is a good indication that the reliability of this study is acceptable.

**Table 3:** Cronbach’s α, composite reliability (CR), average variance extracted (AVE) and Rho-A values for the construct.

Variable	Cronbach’s α	CR	AVE	Rho-A
Functional value for money	0.974	0.981	0.927	0.974
Perceived product quality	0.977	0.983	0.936	0.977
Perceived product scarcity	0.973	0.982	0.948	0.973
Functionality of product design	0.966	0.975	0.908	0.967
Instant feedback on product information	0.983	0.987	0.951	0.983
Perceived product knowledge of streamers	0.972	0.981	0.946	0.972
Product affective involvement	0.969	0.980	0.941	0.969
Product cognitive involvement	0.975	0.983	0.952	0.975
Felt urge to buy impulsively	0.969	0.980	0.942	0.969
Impulse buying behavior	0.980	0.985	0.944	0.980

##### 4.1.1.2. Validity analysis

In this paper, the validity of this measurement is examined in terms of convergent validity and discriminant validity. As seen in Table 1, the majority of factor loading coefficients for the items of the measurement model exceeded 0.7, and as seen in Table 3, the AVE values for all constructs exceeded the threshold of 0.5, both of which are strong indications that the measurement model had good convergent validity.

As shown in Table 4, the square root of the AVE values for all constructs is greater than the absolute value of the correlation coefficients constructed in their corresponding rows and columns, while Table 5 shows that the HTMT ratio estimates are all below 0.90, indicating that the observed variables effectively reflect their latent variables, and that the latent variables have good discriminant validity.

**Table 4:** Means, standard deviations, and correlation coefficients of the constructs.

Variable	Mean	SD	PRI	PQ	PST	PDF	IF	KN	CI	AI	BI	IB
Functional value for money	4.467	1.980	**0.971**									
Perceived product quality	4.488	1.996	0.554^**^	**0.976**								
Perceived product scarcity	4.395	2.098	0.496^**^	0.564^**^	**0.972**							
Functionality of product design	4.462	1.939	0.433^**^	0.514^**^	0.504^**^	**0.970**						
Instant feedback on product information	4.349	2.049	0.474^**^	0.511^**^	0.522^**^	0.453^**^	**0.975**					
Perceived product knowledge of streamers	4.439	2.020	0.414^**^	0.489^**^	0.419^**^	0.414^**^	0.377^**^	**0.973**				
Product cognitive involvement	4.545	1.979	0.533^**^	0.537^**^	0.533^**^	0.535^**^	0.506^**^	0.441^**^	**0.974**			
Product affective involvement	4.785	1.823	0.441^**^	0.497^**^	0.496^**^	0.426^**^	0.451^**^	0.420^**^	0.362^**^	**0.963**		
Felt urge to buy impulsively	4.794	1.829	0.373^**^	0.382^**^	0.409^**^	0.411^**^	0.386^**^	0.331^**^	0.466^**^	0.400^**^	**0.967**	
Impulse buying behavior	5.227	1.552	0.414^**^	0.422^**^	0.425^**^	0.423^**^	0.394^**^	0.330^**^	0.438^**^	0.455^**^	0.371^**^	**0.953**

Mean, mean; SD, standard deviation; the diagonally bolded numbers indicate the square root of the AVE value. ***p < 0.001,**p < 0.01, *p < 0.05.

**Table 5:** HTMT ratio estimates.

Variable	PRI	PQ	PST	PDF	IF	KN	CI	AI	BI	IB
Functional value for money										
Perceived product quality	0.570									
Perceived product scarcity	0.509	0.577								
Functionality of product design	0.447	0.528	0.517							
Instant feedback on product information	0.486	0.522	0.531	0.464						
Perceived product knowledge of streamers	0.426	0.503	0.430	0.426	0.385					
Product cognitive involvement	0.549	0.551	0.545	0.551	0.518	0.454				
Product affective involvement	0.454	0.510	0.508	0.439	0.461	0.432	0.372			
Felt urge to buy impulsively	0.383	0.391	0.418	0.423	0.394	0.339	0.478	0.410		
Impulse buying behavior	0.427	0.434	0.436	0.437	0.404	0.340	0.451	0.469	0.382	

#### 4.1.2. Structural model

The evaluation of the structural model focused on testing the validity of the structural model and evaluating whether the causal relationships defined in the theory construction phase hold.

##### 4.1.2.1. Validity test

As suggested by Hair et al. (2021), when $R^{2}$ is greater than 0.26, the explanatory power of the theoretical model is considered to be good. The predictive relevance of the statistical model is measured by *Q*^2^ and if the predictive value is greater than 0, then the model has good predictive power. As seen in Table 6, the model in this study clearly had a strong predictive power. For the effect size *f*^2^, in general, 0.02, 0.15, and 0.35 are considered to be the thresholds for small, medium, and high effect sizes, while there are also numerous studies (e.g., Reilly et al., 2007; Hagerty et al., 2020; Rodrigues et al., 2020) that consider the *f*^2^ to be acceptable as long as it exceeds 0.01. Therefore, the effect sizes of the paths in this study were deemed acceptable except for the effect size of 0.009, which is <0.01, for the effect of the functionality of product design on the product affective involvement.

**Table 6:** Predictive power of constructs.

Implicit variable	*R* ^2^	*Q* ^2^	Independent variable	Effect (*f^2^*)
Product cognitive involvement	0.474	0.442	Functional value for money	0.043
			Perceived product quality	0.013
			Perceived product scarcity	0.023
			Functionality of product design	0.053
			Instant feedback on product information	0.025
			Perceived product knowledge of streamers	0.013
Product affective involvement	0.371	0.338	Functional value for money	0.011
			Perceived product quality	0.018
			Perceived product scarcity	0.030
			Functionality of product design	0.009
			Instant feedback on product information	0.018
			Perceived product knowledge of streamers	0.020
Felt urge to buy impulsively	0.279	0.258	Product cognitive involvement	0.165
			Product affective involvement	0.086
Impulse buying behavior	0.304	0.272	Product cognitive involvement	0.078
			Product affective involvement	0.110
			Felt urge to buy impulsively	0.015

##### 4.1.2.2. Overall model testing

Predicting the fitness of a model is usually judged using the global criterion of Goodness of Fit (GoF). The GoF value for this study was calculated as 0.579, which also fully indicates that the model was well adapted.

##### 4.1.2.3. Path coefficient test

**Figure 2:**
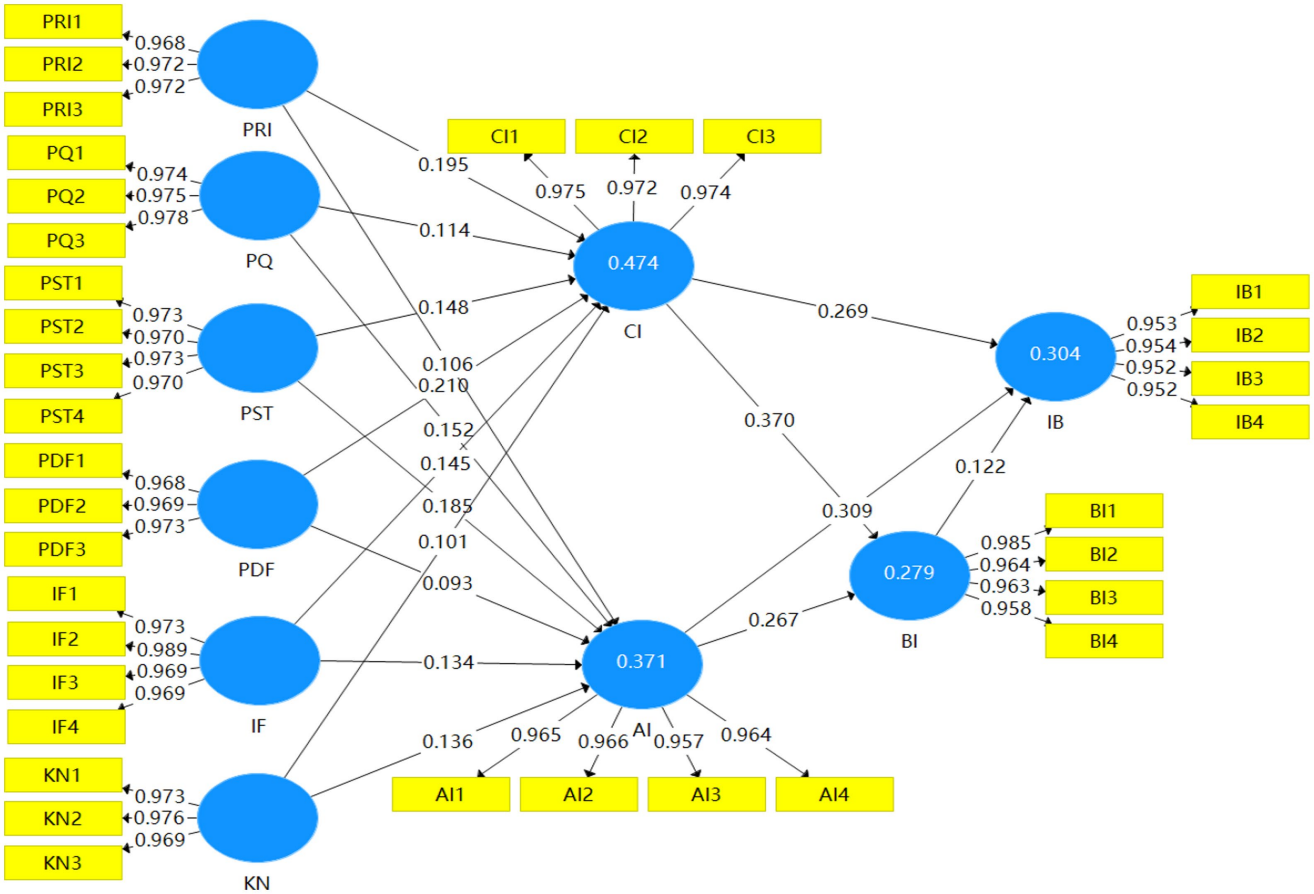
Structural model results.

The model was well fitted by a standardized root mean square residual (SRMR) of 0.015 (<0.08), a difference distance between dULS and dG of less than the HI 95% bootstrap value, and an NFI of 0.950 (>0.9). Table 7 summarizes the empirical results of the study hypotheses, and the results are shown in Figure 2.

**Table 7:** Hypothesis testing (*N* = 504).

Hypothesis	Path	Beta coefficient	*T*-value	*p*-value	Result
*H1*-a	Functional value for money → Product cognitive involvement	0.195	3.950	0.000	Accepted
*H1*-b	Functional value for money → Product affective involvement	0.106	2.008	0.045	Accepted
*H2*-a	Perceived product quality → Product cognitive involvement	0.114	2.292	0.022	Accepted
*H2*-b	Perceived product quality → Product affective involvement	0.152	2.663	0.008	Accepted
*H3*-a	Perceived product scarcity → Product cognitive involvement	0.148	2.712	0.007	Accepted
*H3*-b	Perceived product scarcity → Product affective involvement	0.185	3.292	0.001	Accepted
*H4*-a	Functionality of product design → Product cognitive involvement	0.210	4.738	0.000	Accepted
*H4*-b	Functionality of product design → Product affective involvement	0.093	1.742	0.082	Declined
*H5*-a	Perceived product knowledge of the streamer → Product cognitive involvement	0.145	3.182	0.001	Accepted
*H5*-b	Perceived product knowledge of the streamer → Product affective involvement	0.134	2.614	0.009	Accepted
*H6*-a	Instant feedback on product information → Product cognitive involvement	0.101	2.151	0.032	Accepted
*H6*-b	Instant feedback on product information → Product affective involvement	0.136	2.856	0.004	Accepted
*H7*-a	Product cognitive involvement → Felt urge to buy impulsively	0.370	8.826	0.000	Accepted
*H7*-b	Product affective involvement → Felt urge to buy impulsively	0.269	5.960	0.000	Accepted
*H8*-a	Product cognitive involvement → Impulse buying behavior	0.267	6.304	0.000	Accepted
*H8*-b	Product affective involvement → Impulse buying behavior	0.309	6.385	0.000	Accepted
*H9*	Felt urge to buy impulsively → Impulse buying behavior	0.122	2.329	0.020	Accepted

The findings indicated that the functional value for money was positively related to product cognitive involvement (*β* = 0.195, *t* = 3.950) and product affective involvement (*β* = 0.106, *t* = 2.008), supporting *H1*-a and *H1*-b. Perceived product quality had a significant effect on product cognitive involvement (*β* = 0.114, *t* = 2.292) and product affective involvement (*β* = 0.152, *t* = 2.663), supporting *H2*-a and *H2*-b. Perceived product scarcity had a positive effect on product cognitive involvement (*β* = 0.148, *t* = 2.712) and product affective involvement (*β* = 0.185, *t* = 3.292), confirming hypotheses *H3*-a and *H3*-b. Functionality of product design had a positive effect on product cognitive involvement (*β* = 0.210, *t* = 4.738), but not a significant effect on product affective involvement (*β* = 0.093, *t* = 1.742), supporting hypothesis *H4*-a and rejecting *H4*-b. Perceived product knowledge of the streamer had a positive effect on product cognitive involvement (*β* = 0.145, *t* = 3.182) and product affective involvement (*β* = 0.134, *t* = 2.614), supporting hypotheses *H5*-a and *H5*-b. Instant feedback on product information had a positive effect on both product cognitive involvement (*β* = 0.101, *t* = 2.151) and product affective involvement (*β* = 0.136, *t* = 2.856), supporting hypotheses *H6*-a and *H6*-b. Product cognitive involvement (*H7*-a; *β* = 0.370, *t* = 8.826), and product affective involvement (*H7*-b; *β* = 0.269, *t* = 5.960) both had a positive effect on felt urge to buy impulsively. Both product cognitive involvement (*β* = 0.267, *t* = 6.304) and product affective involvement (*β* = 0.309, *t* = 6.385) had a positive effect on impulse buying behavior, supporting hypotheses *H8*-a and *H8*-b. Finally, felt urge to buy impulsively was positively related to impulse buying behavior (*β* = 0.122, *t* = 2.329), supporting hypothesis *H9*.

Meanwhile, in calculating the *R*^2^, the structural model of this study explained 47.4% of product cognitive involvement, 37.1% of product affective involvement, 27.9% of the felt urge to buy impulsively, and 30.4% of impulse buying behavior.

In summary, of the 17 hypotheses proposed in this study, one was not confirmed by the data (see Figure 2), while the remaining 16 were verified to varying degrees. Also, the results of the study indicate that product cognitive involvement, product affective involvement, felt urge to buy impulsively, and impulse buying behavior all have a high amount of explained variance, confirming the good predictive power of the research model.

### 4.2. Artificial neural network

**Figure 3:**
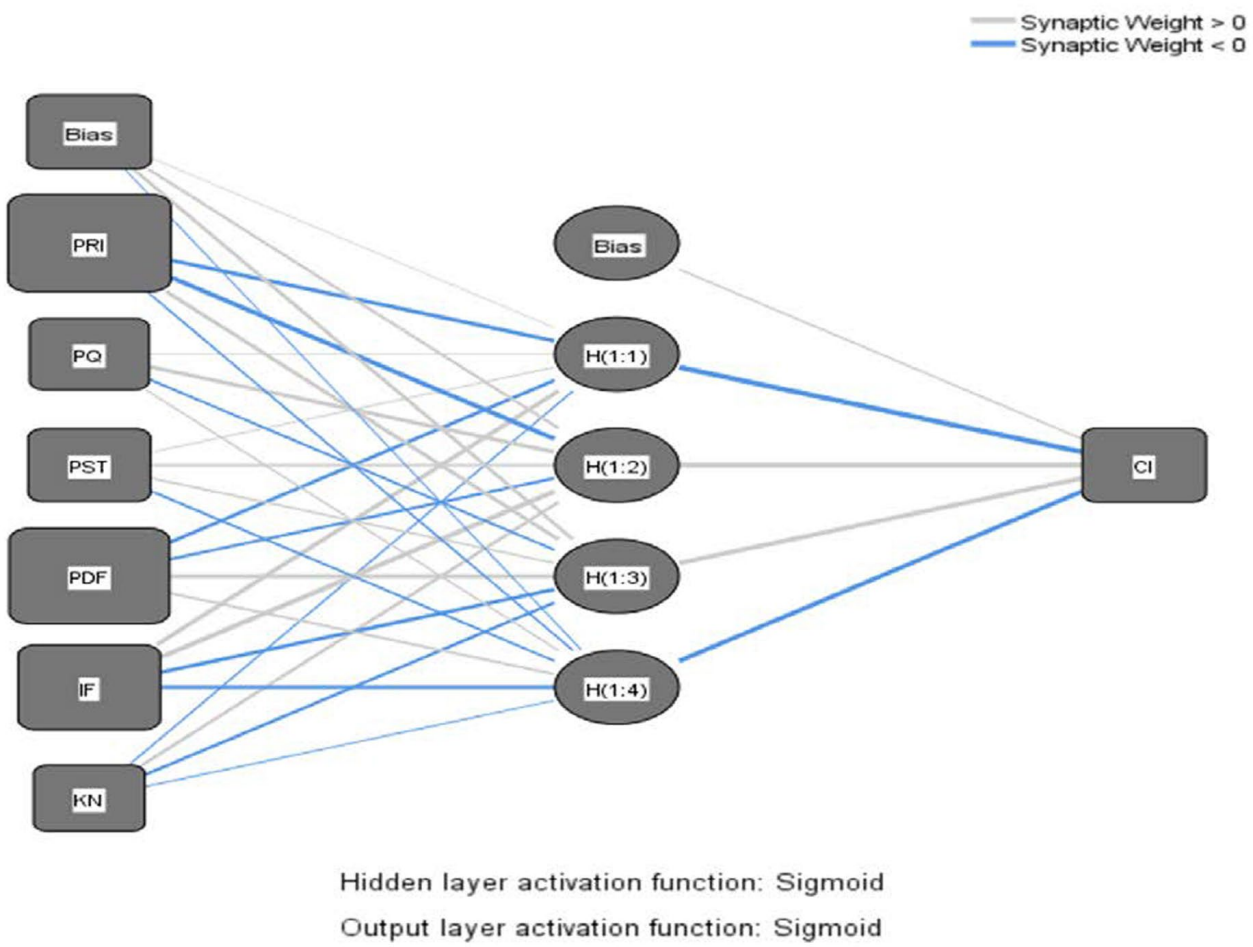
Neural network model – product cognitive involvement.

**Figure 4:**
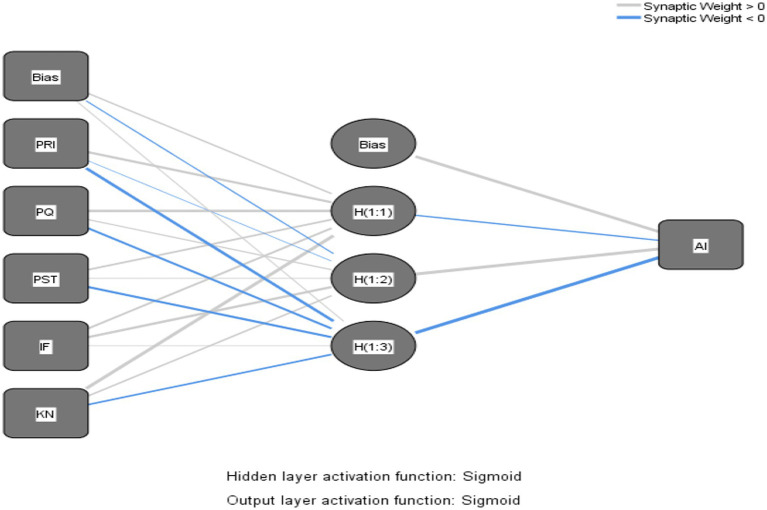
Neural network model – product affective involvement.

**Figure 5:**
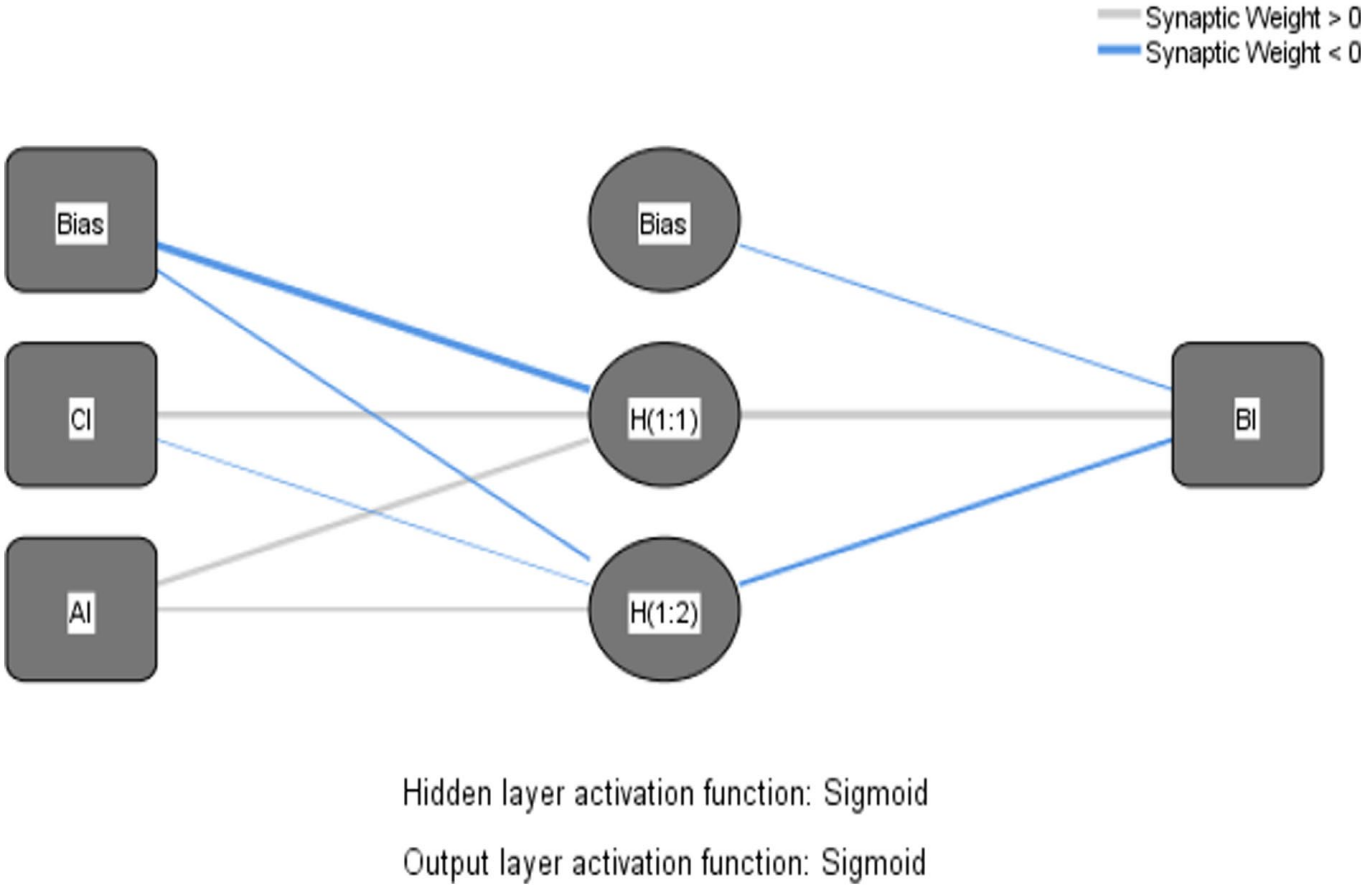
Neural network model – felt urge to buy impulsively.

**Figure 6:**
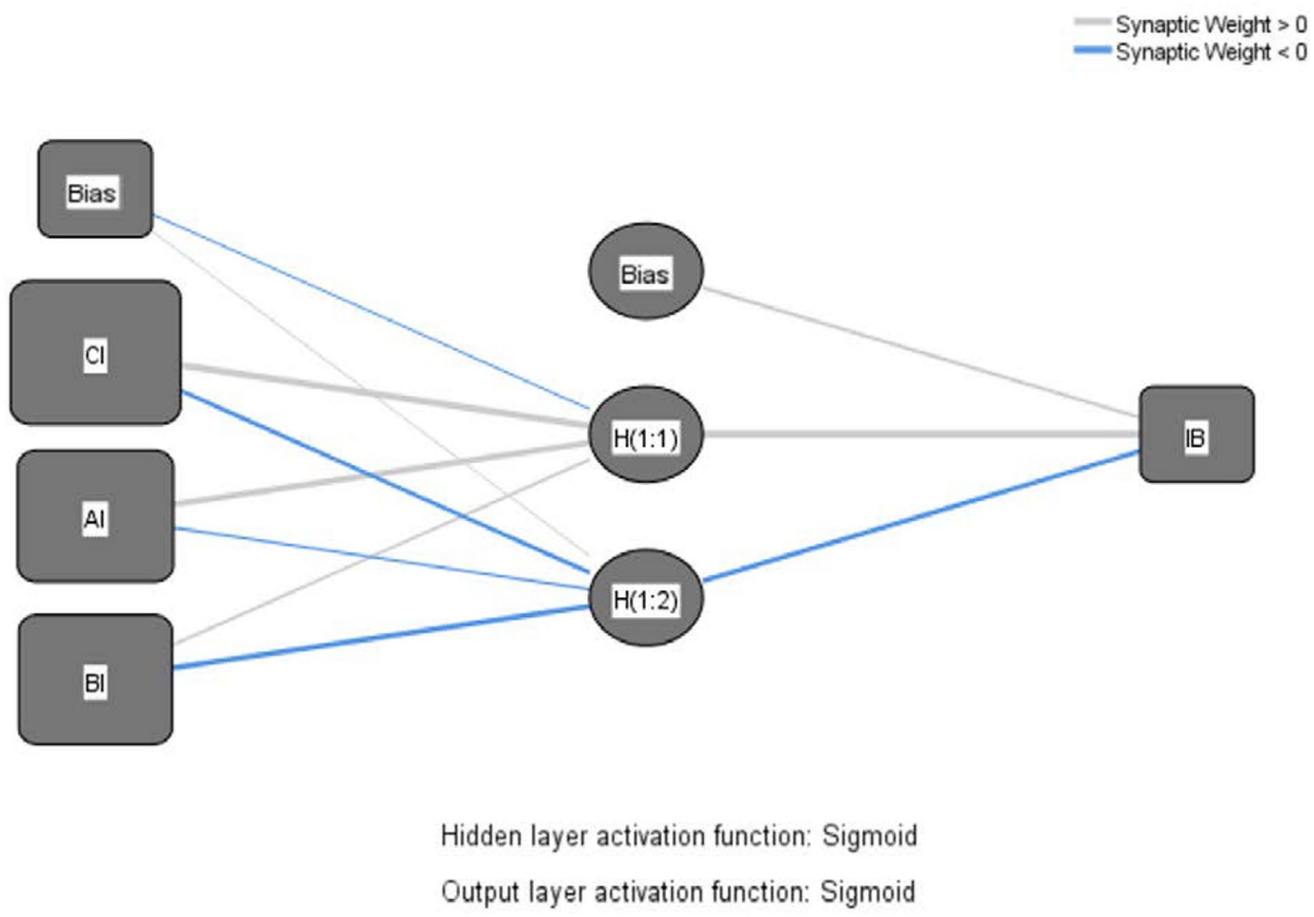
Neural network model – impulse buying behavior.

Similar to Leong et al. (2013), in the current paper, paths with significant effects in SEM were included as input neurons in the neural network analysis, which can be decomposed into four ANN models as shown in the Neural Network Models, Figures 3–6.

The Sigmoid function was also used as the activation function for the input and hidden layers (Sharma et al., 2017). Ten networks were generated for each ANN model and a tenfold cross-validation routine was used to avoid overfitting, with 90% of the data used for training and 10% for prediction (Ooi et al., 2018), and the root mean square error (RMSE; Lee et al., 2020) was obtained from the training and testing process. As shown in Table 7, the average RMSE values were 0.1714 (*SD* = 0.0082) and 0.1646 (*SD* = 0.0064) for training and testing in Model 1, 0.1703 (*SD* = 0.0035) and 0.1683 (*SD* = 0.0128) for training and testing in Model 2, and 0.1703 (*SD* = 0.0035) and 0.1683 (*SD* = 0.0128) for training and testing in model 3. RMSE values were 0.1900 (*SD* = 0.0051) and 0.1807 (*SD* = 0.0145), respectively, and the average RMSE values for training and testing in model 4 were 0.1585 (*SD* = 0.0028) and 0.1569 (*SD* = 0.0101), respectively. The RMSE means for both processes were relatively small (Table 8), indicating that ANNs could better calibrate the data and have a higher level of predictive accuracy (Ooi et al., 2018).

**Table 8:** Root mean square error values for artificial neural networks.

Artificial neural network	Model 1 input: PRI, PQ, PST, PDF, IF, KN output: CI		Model 2 input: PRI, PQ, PST, IF, KN output: AI		Model 3 input: CI, AI output: BI		Model 4 input: CI, AI, BI output: AI	
	Training	Test	Training	Test	Training	Test	Training	Test
1	0.1638	0.1688	0.1720	0.1696	0.1876	0.178	0.1611	0.1533
2	0.1754	0.1641	0.1716	0.1674	0.1904	0.1866	0.1543	0.1697
3	0.1674	0.1588	0.1667	0.1748	0.1927	0.1753	0.1559	0.1588
4	0.1624	0.1777	0.1687	0.1669	0.1925	0.1611	0.1583	0.1499
5	0.1803	0.1609	0.1769	0.1382	0.1899	0.1635	0.1546	0.1689
6	0.1805	0.1640	0.1713	0.1759	0.1852	0.1951	0.1619	0.1478
7	0.1586	0.1693	0.1729	0.1876	0.2018	0.1937	0.1582	0.1410
8	0.1716	0.1606	0.1686	0.1624	0.1851	0.1940	0.1612	0.1597
9	0.1822	0.1550	0.1696	0.1654	0.1850	0.1984	0.1602	0.1499
10	0.1718	0.1666	0.1644	0.1746	0.1899	0.1649	0.1591	0.1695
Mean	0.1714	0.1646	0.1703	0.1683	0.1900	0.1807	0.1585	0.1569
SD	0.0082	0.0064	0.0035	0.0128	0.0051	0.0145	0.0028	0.0101

Furthermore, based on the proposed method of Leong et al. (2019), we calculated a goodness-of-fit coefficient similar to $R^{2}$ in the PLS-SEM analysis to further evaluate the performance of the ANN model. The results showed that the input neuron nodes could predict 98.92, 98.48, 98.39, and 97.28% of the variance of product cognitive involvement, product affective involvement, felt urge to buy impulsively, and impulse buying behavior, respectively. The *R*^2^ values obtained in the ANN analysis were significantly higher than those obtained in the PLS-SEM analysis, which also indicates that the endogenous structure was better in the ANN analysis interpretation, demonstrating the deep learning architecture with two hidden layers and the ability of ANN in fitting nonlinear relationships.

In addition, this paper used sensitivity analysis to measure the relative importance of the predictor variables, as shown in Table 9. The normalized importance of all input neurons was calculated by dividing the average importance with the highest importance and expressing it as a percentage. In the order of the normalized importance of ANN and SEM path coefficients, the most important influencing variables in Models 1 and 2 remained consistent, while Models 3 and 4 were identical, indicating the robustness of the overall analysis results. Inconsistent with SEM, in the ANN analysis, in Model 1, perceived product scarcity was ranked third from the last position, while the last three positions in Model 2 changed from perceived product knowledge of streamers, instant feedback on product information, and functional value for money to functional value for money, instant feedback on product information, and perceived product knowledge of streamers. This may be due to the higher predictive accuracy of ANN and the linear and non-linear relationships between the predictor variables (Oparaji et al., 2017). That is, in Model 1, after predicting the non-linear relationship between perceived product scarcity and product cognitive involvement, perceived product scarcity actually had less impact than instant feedback on product information, perceived product quality, and perceived streamer product knowledge. In Model 2, the ranking of importance changed after predicting a non-linear relationship between perceived streamer product knowledge, instant feedback on product information, and functional value for money and perceived product cognitive involvement. This fact would have been masked if only a single-stage analysis (i.e., PLS-SEM) had been used, so this demonstrates the value of the two-stage analysis approach (SEM-ANN) in this study.

**Table 9:** Sensitivity analysis of neural networks.

Neural network	Model 1				Model 2				Model 3				Model 4			
	PRI	PQ	PST	PDF	IF	KN	PRI	PQ	PST	IF	KN	CI	AI	CI	AI	BI
1	1.000	0.442	0.478	0.967	0.734	0.333	0.809	1.000	0.917	0.845	0.924	1.000	0.929	1.000	0.873	0.852
2	0.701	0.800	0.650	0.853	1.000	0.779	0.558	0.629	1.000	0.989	0.829	1.000	0.820	0.761	1.000	0.589
3	0.917	0.574	0.699	1.000	0.791	0.595	1.000	0.955	0.860	0.750	0.789	1.000	0.814	0.623	1.000	0.400
4	1.000	0.589	0.683	0.999	0.580	0.581	0.819	0.838	1.000	0.570	0.860	1.000	0.905	0.852	1.000	0.504
5	0.971	0.781	0.414	0.362	1.000	0.675	0.495	0.749	1.000	0.350	0.376	0.838	1.000	0.610	1.000	0.484
6	0.997	1.000	0.461	0.907	0.631	0.076	0.653	0.831	1.000	0.811	0.694	1.000	0.802	0.883	1.000	0.490
7	1.000	0.685	0.361	0.886	0.629	0.398	0.717	0.549	1.000	0.491	0.474	0.972	1.000	0.727	1.000	0.447
8	0.899	0.290	0.501	1.000	0.740	0.490	0.671	0.770	1.000	0.725	0.638	1.000	0.795	0.831	1.000	0.496
9	0.554	0.522	0.111	0.612	0.804	1.000	0.726	1.000	0.864	0.750	0.619	1.000	0.815	0.733	1.000	0.368
10	0.432	0.362	0.586	1.000	0.559	0.473	0.733	0.658	1.000	0.894	0.558	1.000	0.748	0.896	1.000	0.393
Average relative importance/percentage	0.847	0.605	0.494	0.859	0.747	0.540	0.718	0.798	0.964	0.718	0.676	0.981	0.863	0.792	0.987	0.502
Normalized relative importance/percentage	98.66	70.41	57.58	100.00	86.98	62.89	74.48	82.76	100.00	74.42	70.13	100.00	87.95	80.18	100.00	50.88

## 5. Discussion

### 5.1. Conclusion

This study used a two-stage SEM-ANN analysis based on product involvement theory to analyze the determinants of impulse buying by livestreaming commerce consumers from the perspective of product features. It found that:

Among the six main antecedents influencing product cognitive involvement, functional value for money, perceived product quality, perceived product scarcity, functionality of product design, instant feedback of product information, and perceived product knowledge of the streamer, all had a positive impact on product cognitive involvement in the SEM-ANN two-stage analysis. Specifically, the functionality of the product design enabled consumers to perceive the usefulness of the product, which meant that it generated stronger product cognitive involvement, which is also consistent with the findings of Rietz et al. (2019). The level of functional value for money was closely related to the utilitarian value of the product, also as in the study of Singh et al. (2021), which establishes a link between functional value for money and product cognitive involvement. Extending the findings of the study by Akram et al. (2018), the positive effect of perceived product scarcity on product cognitive involvement was further demonstrated. Interestingly, perceived product scarcity moved from being ranked in the third position among all influencing variables in the SEM analysis to the last position in the ANN analysis, which may be explained by the non-linear nature and higher predictive accuracy of the ANN model. Instant feedback of product information is also considered to be the main difference between livestreaming commerce and other forms of commerce, where consumers may receive the desired product information through real-time interactions, gaining perceptions of the product usefulness, and achieving cognitive product involvement. In contrast to existing studies exploring the effect of consumer involvement on perceived product quality (e.g., Tsiotsou, 2006; Campbell et al., 2014; Rokonuzzaman et al., 2020), this study demonstrates the effect of perceived product quality on consumer involvement, meaning that consumers perceive that the higher the quality of the product recommended by the streamer, the more they will perceive cognitive involvement regarding the usefulness of the product. The perceived product knowledge of the streamer increases the consumer’s recognition of the streamer’s expertise and increases the perceived involvement in the product, which is also consistent with the study of Chen et al. (2022).

Among the six independent variables influencing product affective involvement, functional value for money, perceived product quality, perceived product scarcity, instant feedback on product information, and perceived product knowledge of streamers had a positive effect on product affective involvement, but no effect of functionality of product design on product affective involvement was found. Perceived product scarcity was shown to be the first variable to have an impact on product affective involvement, serving as a common marketing strategy and becoming an effective consumer pursuit of uniqueness (Wu et al., 2012), which may also affect consumers’ competitive perceptions (Oruc, 2015). Perceived product quality is an influence factor of the trust and consumer satisfaction (e.g., Gök et al., 2019), which means that if a consumer perceives higher product quality, this may provoke the expected emotion of using the product and trigger affective involvement. Functional value for money rose from the last position in the SEM analysis to the third position in the ANN analysis (relative importance: 74.84%); it is possible that consumers who intend to shop live are themselves relatively price sensitive with regards to the product and would prefer to buy products that are good value for money. Interestingly, instant feedback on product information and perceived product knowledge of the streamer were least influential in product affective involvement; it is possible that livestreaming commerce consumers are more inclined to seek product-related information through immediate communication than achieving self-satisfaction through the communication itself. At the same time, the streamer’s product knowledge will often give consumers more practical information (Chen et al., 2022) rather than hedonic information. Surprisingly, the functionality of the product design had no effect on the product affective involvement, contradicting the studies of Homburg et al. (2015) and Reimann et al. (2010), possibly because consumers experience the usefulness of a product more through its features and are not necessarily able to develop a perception of the product that they find as interesting.

Among the two main antecedents affecting consumers’ felt urge to buy impulsively, both product cognitive involvement and product affective involvement had a significant impact. The influence of product cognitive involvement on consumers’ felt urge to buy impulsively ranked first, while the relative importance of product affective involvement on consumers’ felt urge to buy impulsively was 87.95%, further supporting the findings of Chan et al. (2017) who noted that online impulse buying organism (affective and cognitive reaction) have a positive impact on online impulse buying response (felt urge to buy impulsively and online impulse buying behavior).

Among the three main antecedents that influence consumers’ impulse buying behavior, product cognitive involvement (relative importance: 80.18%), product affective involvement (relative importance: 100%), and felt urge to buy impulsively (relative importance: 50.88%) all had significant effects on impulse buying behavior. In other words, consumers who perceived the usefulness of a product recommended by the streamer were more likely to immediately purchase a product they did not intend to buy. Product affective involvement, on the other hand, led to a stronger connection with the streamer-recommended product and impulse buying behavior, which is also consistent with the findings of Chan et al. (2017). The impact of felt urge to buy impulsively on impulse buying behavior has already been proven by numerous studies (Shen and Khalifa, 2012; Zhao et al., 2019), and in a livestreaming commerce environment, the probability of impulse buying behavior arising if the consumer feels an urge to buy impulsively is even higher.

### 5.2. Theoretical contribution

The main theoretical contributions of this study are, first, that use of a two-stage SEM-ANN predictive analysis can provide a more comprehensive understanding of the relationships between the variables and provide important methodological contributions from a statistical perspective. ANN analysis can compensate for the weakness of linear SEM analysis, opening up new perspectives in the study of understanding the impact of product features on consumer impulse buying.

Second, the study constructed a theoretical model of impulsive purchase of livestreaming commerce consumers from the perspective of product features, which provides empirical support for product involvement theory in a livestreaming scenario. Compared with existing studies which have looked at the characteristics of livestreaming platforms (Gong et al., 2020), streamer characteristics (Park and Lin, 2020; Ma, 2021), and social presence (Ming et al., 2021), this paper focused on the important factor of product characteristics, which effectively explains the effectiveness of impulse buying of livestreaming commerce consumers and broadens the application context of product involvement theory.

Third, this study provides another possible explanation for a widely accepted view in the existing literature. Specifically, this study suggests that the effects of perceived product quality, perceived streamers product knowledge, functional value for money, perceived product quality on product cognitive involvement, instant feedback on product information on product affective involvement, and felt urge to buy impulsively on impulse buying behavior may be relatively subtle, and that functionality of product design do not necessarily affect livestreaming commerce consumers’ affective involvement with products. These relationships have been more or less proven in previous studies, however, this study provides an alternative explanation of the relationship between these variables that may have some theoretical implications for other related studies.

### 5.3. Practical implications

This paper does have some practical value. First, livestreaming commerce should determine a reasonable product selling price, and allow consumers to form their own perceptions of whether the products are value for money through promotional means such as seconds, giving out red packets, and coupons. Second, livestreaming commerce should effectively demonstrate product quality, such as by providing quality inspection reports, celebrity endorsements, or expert certification to change the inherent bias of consumers that products sold live are of poorer quality. Third, livestreaming commerce should focus on the product’s scarcity due to limited time or through a marketing strategy highlighting its limited nature so that consumers perceive the product to be useful, effective, and interesting to stimulate consumers’ impulse to buy. Fourth, the context of live products should be as rich as possible, and the streamer should show the multi-purpose nature of products in full so that consumers recognize the usefulness of the product. Fifth, livestreaming commerce should respond to consumer questions in a timely manner during livestreaming, as it can answer consumers’ questions about the product, as well as stimulating other consumers to buy *via* the herd effect. Finally, livestreaming commerce should allow consumers to form the perception that the streamer is an expert on the product, so that they believe in the streamers professionalism and immediately buy the recommended product.

### 5.4. Research limitations and future prospects

This study inevitably has certain limitations and provides ideas for future research. First, due to the ease of online data collection and the specificity of the study population, this study used a cross-sectional design to analyze the correlational relationships among the study variables. Although this method is commonly used in empirical research, it cannot fully determine the direction of the variable relationships. Future research should adopt experimental studies to explore causal inference. Second, while we identified the important variables to be explored through an extensive review of the relevant literature, this study may have missed other variables (e.g., trust in the product, convenience, perceived risk, relationship strength). Future research should integrate these other variables to develop a more comprehensive view of impulse buying by live consumers from the perspective of product characteristics. Third, this study did not consider boundary conditions, nor did it include other possible moderating variables such as cultural context and personal traits which should also be incorporated into the theoretical model of the study in the future. Finally, with regards to the ease of data collection, this study conducted data collection using an online platform, which may not be fully representative of the entire livestream consumer population. Future research should focus on finding a suitable sampling approach to better understand the purchasing behavior of livestreaming consumers.

## Data availability statement

The raw data supporting the conclusions of this article will be made available by the authors, without undue reservation.

## Ethics statement

The studies involving human participants were reviewed and approved by School of Management at Guizhou University. The ethics committee waived the requirement of written informed consent for participation.

## Author contributions

XG designed the research framework and wrote the paper. XJ analyzed the data. XG edited the paper. All authors contributed to the article and approved the submitted version.

## Funding

Project supported by the Humanities and Social Sciences Research Youth Program of Guizhou University (Grant No. GDQN2022002), the Talent Introduction Program of Guizhou University (Grant No. 2021035), the Key Research Base of Humanities and Social Sciences of Guizhou Provincial Department of Education, the Talent Introduction Program of School of Management at Guizhou University (Grant No. 22GLR001), and the Guizhou Provincial Science and Technology Projects (Grant No. ZK2021G339, 2022G080).

## Conflict of interest

The authors declare that the research was conducted in the absence of any commercial or financial relationships that could be construed as a potential conflict of interest.

## Publisher’s note

All claims expressed in this article are solely those of the authors and do not necessarily represent those of their affiliated organizations, or those of the publisher, the editors and the reviewers. Any product that may be evaluated in this article, or claim that may be made by its manufacturer, is not guaranteed or endorsed by the publisher.

## Footnotes

^1^www.credamo.com
